# The Use of CytoSorb Therapy in Critically Ill COVID-19 Patients: Review of the Rationale and Current Clinical Experiences

**DOI:** 10.1155/2021/7769516

**Published:** 2021-07-17

**Authors:** Juan Carlos Ruiz-Rodríguez, Zsolt Molnar, Efthymios N. Deliargyris, Ricard Ferrer

**Affiliations:** ^1^Department of Intensive Care, Hospital Universitari Vall d'Hebron, Shock Organ Dysfunction and Resuscitation Research Group, Vall d'Hebron Institut de Recerca (VHIR), Barcelona, Spain; ^2^CytoSorbents Europe GmbH, Berlin, Germany; ^3^Institute for Translational Medicine, School of Medicine, University of Pécs, Pécs, Hungary; ^4^Department of Anesthesiology and Intensive Therapy, Poznan University of Medical Sciences, Poznan, Poland; ^5^Department of Anesthesiology and Intensive Therapy, Semmelweis University, Budapest, Hungary; ^6^CytoSorbents Corporation, Monmouth Junction, NJ, USA

## Abstract

The COVID-19 pandemic has led to the biggest global health crisis of our lifetime. There is accumulating evidence that a substantial number of critically ill COVID-19 patients exhibit a dysregulated host response manifesting as cytokine storm or cytokine release syndrome, which in turn contributes to the high observed rates of mortality. Just as in other hyperinflammatory conditions, extracorporeal cytokine removal may have potential beneficial effects in this subgroup of COVID-19 patients. The CytoSorb blood purification device is the most extensively investigated cytokine removal platform with considerable evidence suggesting that early intervention can provide rapid hemodynamic stabilization and improvement in vital organ functions. The purpose of this review is to provide an overview of the pathophysiological background of hyperinflammation in COVID-19 and to summarize the currently available evidence on the effects of hemoadsorption in these patients.

## 1. Background

The COVID-19 pandemic has led to the biggest global health crisis of our lifetime, particularly in intensive care units (ICUs) [1]. The disease has caused not only high infectivity and fatality but also universal economic burden and heavy financial losses [2]. As per the latest World Health Organization (WHO) consensus data (website accessed 27.04.2021), there have been more than 146 million cases and over 3 million casualties reported worldwide [3].

There is accumulating evidence that a substantial number of critically ill COVID-19 patients frequently exhibit viral RNAemia together with a dysregulated immune response [4] with hyperinflammation manifesting as a cytokine storm or as cytokine release syndrome (CRS), which in turn contributes to the high observed rates of mortality [5, 6]. The cytokine profile in these COVID-19 cases seems to resemble secondary hemophagocytic lymphohistiocytosis (sHLH), a severe hyperinflammatory syndrome, which in nearly 30% of cases stem from a viral infection as the underlying condition [5, 7–9]. Reports from China and Italy showing elevated ferritin levels, a recognized hallmark of HLH, further corroborate the mechanistic similarities with severe COVID-19 cases [10].

The above mechanism supports the hypothesis that extracorporeal cytokine removal may have beneficial effects in COVID-19 patients similar to those seen in other hyperinflammatory conditions [11]. In addition, the high mortality observed with severe COVID-19 disease may at least in part be explained by a differential response of these patients to conventional treatments that have been applied in previous flu epidemics [12, 13]. Accordingly, the recognition that hyperinflammation plays a central role in critical COVID-19 cases coupled with the urgent need for alternative treatment modalities has resulted in thousands of CytoSorb treatments worldwide. The purpose of this review is to summarize the accumulating evidence and clinical results from global experiences with CytoSorb therapy in COVID-19.

## 2. Early Experiences

Following the dramatic increases in critically ill COVID-19 patients that have overwhelmed ICUs around the world, many experts began recognizing some important and distinct features of this novel clinical syndrome. A representative excerpt from an article in the Lancet Respiratory Medicine by Ronco et al. demonstrates the early recognition of the unique pathophysiology and the desire by global experts to attempt new treatment modalities: *“Finally, a sepsis-like syndrome might occur frequently due to the virus itself or to a superimposed bacterial infection and in this case, since pharmacological approaches have shown poor results, new extracorporeal organ support therapies including haemoadsorption and haemoperfusion, with new sorbent cartridges designed to remove cytokines and other circulating mediators, should be considered.”* [14]. Tay et al. further supported this therapeutic approach principle by stating in their elaboration of COVID-19 pathophysiology that “*controlling the inflammatory response may be as important as targeting the virus*” [15].

**Figure 1:**
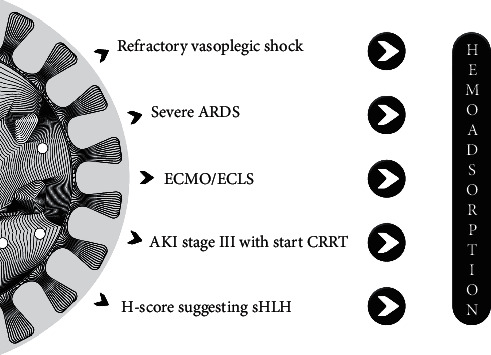
Potential indications for hemoadsorption in COVID-19 patients. ARDS, acute respiratory distress syndrome; ECMO/ECLS, extracorporeal membrane oxygenation/extracorporeal life support; CRRT, continuous renal replacement therapy; HLH, hemophagocytic lymphohistiocytosis.

Several national medical societies incorporated CytoSorb into their treatment guidelines early on during the pandemic (Figure 1) [16]. The Italian Society of Nephrology was one of the first to recommend CytoSorb use in COVID-19 patients with acute kidney injury (AKI) stage 3 receiving continuous renal replacement therapy (CRRT) [17]. The Handbook of COVID-19 Prevention and Treatment from the Zhejiang University School of Medicine, China [18], and a Colombian/Panama guideline also recommended anticytokine storm treatment in the early stage of critical cases [19]. Most importantly, on April 10, 2020, the United States Food and Drug Administration granted Emergency Use Authorization for CytoSorb use in “*critically ill COVID-19 patients with confirmed or imminent respiratory failure*” [20].

## 3. Rationale for CytoSorb Therapy in COVID-19

There are two types of triggers for a host immune response: damage (meaning tissue trauma caused by surgery, de facto trauma, ischemia-reperfusion injury, sterile inflammation, etc.) or pathogen-associated insults. Circulating mediators detected in these scenarios are called damage-associated molecular patterns (DAMP) and pathogen-associated molecular patterns (PAMP), respectively, and both can lead to the systemic release of cytokines and other inflammatory mediators. It has been found that high levels of both pro- and anti-inflammatory mediators are associated with increased mortality [21].

CytoSorb therapy, an adsorptive blood purification technology, designed to eliminate elevated levels of cytokines and other inflammatory mediators from the blood, is intended to serve as an adjunctive therapy in systemic hyperinflammation by modulating the cytokine storm.

The adsorber was originally intended for conditions where cytokine plasma concentrations are increased (e.g., in septic shock or in other noninfectious hyperinflammatory conditions) but has also been shown to be effective for the removal of myoglobin and bilirubin, as well as the antithrombotics ticagrelor and rivaroxaban [22–24]. The adsorber removes predominantly hydrophobic substances with a molecular weight below 60 kDa from whole blood. Its removal properties can be explained by the rather broad spectrum but size-selective binding of the adsorption polymer itself, while there also seems to be a concentration-dependent removal efficiency, where high plasma concentrations of substances are cleared more efficiently than lower levels. Of note, the physicochemical mechanisms protect against the complete removal of cytokines.

Evidence that CytoSorb can effectively remove circulating inflammatory cytokines was initially shown in a randomized septic shock animal experiment [25]. CytoSorb was originally approved in the European Union (EU) in 2011, and since then, over 130,000 treatments have been performed worldwide mainly for management of systemic hyperinflammation and refractory shock. CytoSorb therapy has also been used successfully in documented cases with HLH [26, 27]. Recent recommendations on the management of HLH patients mention cytokine adsorption, which may aid in rescuing critically ill patients from a deleterious cytokine storm [28].

CytoSorb therapy should be seen as an adjunctive therapy to be considered in cases when standard therapy does not achieve sufficient clinical stabilization of the patient [29]. Start of the treatment, however, should still be early, i.e., within the first 24 hours after diagnosis of, e.g., septic shock [30], or development of life-threatening COVID-19 [31]. Each adsorber can be used for a maximum of 24 hr, and therapy with a new adsorber should be continued until sufficient clinical improvement is achieved. The flow rate through the device, which can be used in a stand-alone approach in pure hemoperfusion mode, or via integration into an ECMO or CRRT circuit, is recommended to be between 150 ml/min and 700 ml/min.

Anticoagulation for using CytoSorb therapy is required, as it is for every extracorporeal approach; however, normally it does not need to be adapted specifically for CytoSorb, when used in conjunction with other extracorporeal therapies [32].

Overall clinical experience and published reports indicate that CytoSorb therapy is frequently associated with rapid hemodynamic stabilization indicated by a reduction in vasopressor needs, accelerated reduction in serum lactate levels, and improvement in lung function, all of which are considered critical clinical outcomes. It is also important to note that the safety of the device is favorable and supported by the fact that there has not been any confirmed unanticipated device-related adverse events reported to date [29, 33, 34].

Although it has been intensively debated in the literature as to whether hyperinflammation is a typical feature of COVID-19 or not, the answer is more complex than just a simple yes or no. Early reports emphasized the presence of cytokine storm, based mainly on the observed elevated interleukin- (IL-) 6 levels [35, 36]. Two recent articles compared cytokine levels from critically ill COVID-19 patients to levels seen in patients with sepsis, acute respiratory distress syndrome (ARDS), and other conditions and concluded that cytokine storm is not a typical feature of COVID-19 [37, 38]. However, the pure definitions of “critical illness” [38] or “ARDS” [37] do not necessarily cover the patient population in which cytokine adsorption may be beneficial. Most of the available literature reporting positive outcomes related to CytoSorb therapy have been performed in patients who shared the following clinical features [29, 33, 34, 39]:

- Vasoplegic shock requiring high dose of vasopressors
- Elevated levels of cytokine and/or inflammatory biomarkers
- At least 2 (cardiovascular and respiratory) but more often 3 (including acute renal) or more organ systems failing before the start of therapy

The clinical phenotype of COVID-19 is highly variable ranging from asymptomatic cases to multisystem organ failure [40], a very similar heterogeneity to the clinical manifestations of sepsis and ARDS [41, 42]. Therefore, while it is true that hyperinflammation may not be the main feature in all critically ill COVID-19 patients [37, 38], several authors have observed cytokine storm in some of their ICU patients [43, 44]. This patient population which shows the signs indicated above, and which do not improve after standard medical therapy, could benefit from extracorporeal cytokine removal by CytoSorb.

In addition to cytokines, other inflammatory mediators such as activated complement factors (C3a and C5a) or even substances such as bradykinin may be important factors in the critical COVID-19 phenotypes and may explain the highly variable extrapulmonary manifestations of this complex and still not fully understood disease [45, 46]. In vitro testing with CytoSorb has revealed efficient removal of C3a and C5a [47, 48].

It is well known that cytokine synthesis and secretion is a continuous process in patients, but it is particularly pronounced in the critically ill. There is good evidence from basic research in animals that hemoadsorption not only results in a verifiable reduction in plasma levels of inflammatory mediators but also acts on an even more profound level, namely, attenuation of the NF-kB DNA binding activity, promoting a decrease in the de novo production of cytokines in both liver tissue and neutrophils [49, 50]. Based on this, a longer lasting effect of hemoadsorption on cytokine levels beyond the primary removal can be assumed.

In this complex pathophysiological setting involving multiple mediators and high grade of redundancy, overlap, and/or feedback mechanisms, the broad range of action of CytoSorb therapy that targets removal of a variety of inflammatory substances in addition to cytokines could be—at least theoretically—beneficial [51].

Hypercoagulopathy represents another distinct clinical feature of COVID-19 and is likely to be triggered by vascular endothelial cell injury [52]. In a prospective multicentric study, 26% COVID-19 critically ill patients had venous thromboembolism [53]. Direct viral injury seems to be an important factor; however, inflammatory contributions to the activation of coagulation may also be involved, thereby adding another potential beneficial contribution of therapeutic approaches that target a modulation of dysregulated immune responses, e.g., hemoadsorption.

Overall, the pathophysiology of COVID-19 probably includes several different harmful pathways and as such it is likely that no single therapy can target all of these simultaneously [54]. However, as some of these pathways are likely to be related to hyperinflammation, targeting this process with an adjunctive therapeutic approach such as hemoadsorption in select critically ill COVID-19 patients seems to have a sound pathophysiologic rationale.

## 4. Clinical Data

The amount of published data with CytoSorb therapy in COVID-19 remains limited, as is generally also the case for other adjunctive therapeutic approaches, given that this is still a relatively new field of indication. Many of the institutional experiences have only been presented during educational webinars, while published data have been limited to case reports and case series with the largest experience to date originating from Saudi Arabia [31].

This recently published case series by Alharthy retrospectively analyzed 50 COVID-19 patients with AKI requiring continuous renal replacement therapy (CRRT) also treated with CytoSorb [31]. Comorbidities in this population included septic shock, acute respiratory distress syndrome (ARDS), and CRS. The clinical effects of CytoSorb therapy (in combination with CRRT) were significant reductions in vasopressor needs, Sequential Organ Failure Assessment (SOFA) score, and lactate, IL-6, and ferritin levels, as well as an improvement in the PaO_2_/FiO_2_ ratio. A preliminary analysis of randomized controlled data from Germany in eight COVID-19 patients on veno-venous extracorporeal membrane oxygenation (vv-ECMO) showed that IL-6 reduction was more pronounced in the CytoSorb + ECMO group (*n* = 4) than in the ECMO only group (*n* = 4) despite higher baseline levels [55].

Another observational study from Italy reported on nine consecutive COVID-19 patients with severe pneumonia requiring continuous positive airway pressure [56]. Five of the patients were treated with hemoperfusion using a CytoSorb adsorber, while the remaining four patients were not treated with CytoSorb and served as the control group. The results showed a better clinical course for the CytoSorb-treated patients than in the control patients. All five CytoSorb patients except one survived, and only two of them had to be intubated and mechanically ventilated, while all control patients required intubation and ventilation, and unfortunately died. A small retrospective analysis in four patients with SARS-CoV-2 pneumonia recently reported outcomes in two patients treated with tocilizumab (TCZ) alone and two patients treated with TCZ plus CytoSorb hemoadsorption (HA) [51]. All patients were mechanically ventilated with levels of IL-6, C-reactive protein (CRP), and PaO_2_/FiO_2_ ratio measured before, during, and after treatment. In all patients, the IL-6 increased during the treatment; after its termination, its values sharply decreased only in those treated with HA. Conversely, CRP decreased in all patients; the PaO_2_/FiO_2_ increased in three patients and remained stable in one.

Another recent publication included 22 COVID-19 patients who were put on ECMO due to the severity of their condition, but who were otherwise unselected [57]. The first group of 11 consecutive patients had a CytoSorb adsorber integrated into the ECMO circuit and were then compared with the next 11 patients on ECMO without CytoSorb. Eight patients survived to 60 days in the CytoSorb group (73%), compared with seven patients in the control group (64%).

Two of the authors of this article (RFR and JCR-R) in a high-volume COVID-19 center [58] recently treated six COVID-19 patients with severe ARDS not responding to prone positioning with adjunctive CytoSorb hemoadsorption (unpublished data) [59]. All patients presented with hyperinflammation and hypercytokinemia. Hemoadsorption was performed between the third and fifth day of admission to the ICU, following detection of hypercytokinemia and the poor response to rescue maneuvers for severe hypoxemia. Four patients were additionally treated with tocilizumab, and three of them also received steroids. CytoSorb hemoadsorption was associated with a significant reduction in IL-6 plasma levels and an improvement in oxygenation (PaO_2_/FiO_2_ ratio) and organ dysfunction (SOFA score). Inflammatory biomarkers (CRP, D-dimers, and ferritin) also improved significantly. Mortality was 33.7%. In these cases, CytoSorb hemoadsorption was an effective and safe rescue therapy for COVID-19 patients with refractory acute respiratory failure associated with hyperinflammation and hypercytokinemia.

Based on the available data, the potential indications of hemoadsorption as an adjuvant therapy in COVID-19 patients are depicted in Figure 1.

Comparisons of reported mortalities under CytoSorb therapy with other reports on mortality in COVID-19 patients should be done with caution and are likely to be inconclusive as patient numbers of the listed publications with CytoSorb are often rather small and reported mortality rates in COVID-19 seem to vary a lot. However, CytoSorb therapy was shown to be associated with rapid hemodynamic stabilization and improvement in respiratory function, both of which are indispensable prerequisites for recovery [34, 39, 60].

CytoSorb therapy has a strong safety profile, which is based on high biocompatibility, concentration-dependent removal, which helps prevent complete removal of physiologic mediators, and size selectivity, which excludes larger substances such as albumin, coagulation factors, and immunoglobulins from substantial removal [61–63].

As with every extracorporeal therapy, unwanted drug removal cannot generally be excluded. However, discussing this issue in depth is well beyond the scope of the current article. Nevertheless, as far as currently or previously used therapeutic approaches in COVID-19 are concerned, data on potential removal of hydroxychloroquine and azithromycin by CytoSorb are not available, so dosing should be adapted according to therapeutic drug monitoring wherever possible. Tocilizumab is not expected to be removed, given its large molecular weight of 148 kDa [64].

## 5. Discussion

Despite the lack of large randomized clinical trials, there has been significant worldwide interest in the use of CytoSorb in critically ill COVID-19 patients, and the number of treated patients is constantly increasing. Of 13 reported case series and studies compiled to date, five have already been published in international peer reviewed journals, while for the other cases, the data analysis is either ongoing or the manuscript is already under preparation. The common feature in all reports is that they reveal some degree of benefit from the use of CytoSorb as an adjunctive therapy, and all agree that the therapy is safe and well tolerated.

### 5.1. Target Patient Population

Based on the available published data, it seems that the most frequent indications for CytoSorb treatment in these critically ill COVID-19 patients were hemodynamic instability, need for renal replacement therapy [26, 31–33], and severe ARDS requiring ECMO [55, 65]. Apart from one study in which treatment was started at an earlier stage when patients did not need vasopressors or mechanical ventilation [56], most patients were very sick on study entry. This indicates that physicians usually reach for CytoSorb as an adjuvant therapy in those who are in a critical condition and have not responded to standard medical therapy. Nevertheless, future studies are needed to more precisely define the appropriate patient characteristics and optimal point of time for CytoSorb treatment initiation.

### 5.2. Treatment Application

Of the 153 published CytoSorb-treated COVID-19 patients with available clinical data, CytoSorb was applied together with CRRT in 101 cases [34–43]. Most of the previously mentioned studies have also applied CytoSorb together with CRRT [29, 33, 34]. However, 26 (17%) patients received treatment in the form of hemoperfusion [31, 39, 56, 66]. There is only one study so far that has tested CytoSorb as a hemoperfusion treatment in patients with refractory septic shock [39] and provided results indicating that CRRT should not be considered as a prerequisite for initiation of CytoSorb therapy. It is also important to note that a relatively large proportion of patients (around 10%) received CytoSorb therapy together with ECMO [34, 39], indicating that severe respiratory failure requiring ECMO therapy is also considered by some groups as an indication for CytoSorb therapy in COVID-19 patients. A summary of the potential indications are depicted in Figure 1.

### 5.3. Biomarker Levels

In general, biomarker levels are not as high in critically ill COVID-19 patients as those seen in septic shock or sepsis with ARDS [37, 38], but all authors have reported at least a moderate, if not strong, elevation in CRP, IL-6, and ferritin, which in context with the clinical picture has prompted them to initiate hemoadsorption. However, at the present time, there is no well-defined threshold for biomarkers to inform the start of CytoSorb therapy and it is unclear whether such thresholds will be established in the future since biomarker levels are affected by several factors and most importantly by the individual host response. This is similar to the huge scatter in inflammatory biomarker distribution in other disease states such as septic shock, ARDS, pancreatitis, or trauma where clear thresholds for diagnosis and treatment have also not been defined [67]. Nevertheless, it seems that relatively lower levels of inflammatory biomarkers in COVID-19 patients do not exclude the presence of cytokine storm; hence, patient selection remains a challenge in whom and when to commence cytokine adsorption as an adjuvant therapy.

### 5.4. Outcomes

Six data sets have reported survival, which varies from 50 to 81% [31, 56, 59, 66, 68, 69]. Observed mortality in general was lower than predicted and better than in control patients without CytoSorb [56]. Hemodynamics and oxygenation—in studies where they were analyzed—also improved as an effect of the therapy (Figure 1).

Hemodynamic stabilization has repeatedly been reported as the main feature and benefit of CytoSorb therapy [29, 34], a finding that has been confirmed in the six COVID-19 data sets in which this outcome was evaluated, and an improvement in the patients' hemodynamics was uniformly reported [65, 69–72].

Acute respiratory failure is the most dreaded complication of COVID-19 often requiring invasive mechanical ventilation or even ECMO support. Recent data from the United Kingdom suggests a paradigm shift in respiratory support as experience in the management of these patients increases (ICNARC website accessed 6.11.2020, icnarc.org). Rates of intubation during the first 24 hours of ICU admission have decreased by roughly 50%, and mortality also seems to be substantially reduced (39.4% vs 20.7%, respectively), while the severity, as indicated by the PaO_2_/FiO_2_, remains similar to what it was during the Spring (March–May 2020) outbreak. Nevertheless, mortality in patients on mechanical ventilation remains very high worldwide [73, 74]. In the current CytoSorb case reports and case series, improvement in oxygenation was reported in all seven data sets that investigated this outcome measure [31, 56, 59, 66, 68, 71, 75]. CytoSorb studies outside the domain of COVID-19 have also reported similar improvements in oxygenation and/or hemodynamic stabilization in patients with ARDS requiring ECMO [34, 76, 77]. Overall, existing evidence supports the notion that CytoSorb may be beneficial in COVID-19 patients with severe ARDS including those requiring ECMO support. An overview of the currently available literature is summarized in Table 1.

### 5.5. Limitations

As CytoSorb therapy requires an extracorporeal circuit, it should be considered an invasive intervention with all potential complications involving cannulation of large veins, including short- and long-term adverse events related to the cannulation itself and also to the necessary anticoagulation. Therefore, special attention must be paid for close monitoring of the hemostasis and the patient. The treatment is also costly; hence, application also depends on the budget of the actual hospital and the ICU. Nevertheless, as we already emphasized earlier, treatment-related serious adverse events have not been reported yet, in fact the opposite is true as most studies conclude that the treatment proved to be safe.

## 6. Conclusions

A subset of patients with COVID-19 develop critical illness during their course of disease that resembles the features of hyperinflammatory conditions often seen outside the domain of viral infections. These serious, potentially life-threatening complications are often due to the dysregulated hyperinflammatory host immune response causing vasoplegic shock, severe hypoxemia, and elevated inflammatory marker levels. Fortunately, most patients will respond to standard intensive care treatments. However, a subgroup of patients fail to improve despite adequate supportive therapy and progress to a more severe illness. In this population, the use of extracorporeal cytokine adsorption appears to provide significant benefits. Although several open questions remain, early evidence with the use of CytoSorb in these critical patients is encouraging. Hopefully, more data will soon be available to help us to further define and fine tune the role of CytoSorb in the treatment arsenal against COVID-19-related critical illness.

## Figures and Tables

**Table 1:** Overview of the currently available literature.

Author	Study title details	Type of study	Country	Number of patients	Improvement in hemodynamics	Improvement in oxygenation	Control of inflammatory response	Reference number from manuscript
Alharthy A	Continuous Renal Replacement Therapy with the Addition of CytoSorb® Cartridge in Critically Ill Patients with COVID‐19 plus Acute Kidney Injury: a Case‐Series. Artificial Organs 2021; 45(5):E101-112	Retrospective case series	Saudi Arabia	50		Yes	Yes	31
Berlot G	Effects of Tocilizumab Versus Hemoadsorption Combined with Tocilizumab in Patients with SARS-CoV-2 Pneumonia: Preliminary Results. Int *J* Artif organs 2021; epub	Retrospective case series	Italy	2		Yes	Yes	51
Rieder M et al.	Cytokine Adsorption in Patients with Severe COVID-19 Pneumonia Requiring Extracorporeal Membrane Oxygenation. Crit Care 2020; 24: 435	Randomized control trial—Interim analysis	Germany	4 vs 4			Yes	55
Rampino T et al.	Hemoperfusion with CytoSorb as Adjuvant Therapy in Critically Ill Patients with SARS-CoV2 Pneumonia. Blood Purif 2020; epub	Retrospective case series	Italy	5 of 9 consecutive pts treated with CytoSorb		Yes	Yes	56
Lebreton G et al.	Longitudinal Cytokine Profiling in Severe COVID-19 Patients on ECMO and Hemoadsorption. AJRCCM 2021; 203(11): 1433–5	Prospective case series	France	11 consecutive patients on CytoSorb compared to 11 noncontemporaneous pts			Yes	57
Ferrer R	Regain control of Inflammation – IL6 Blockers or CytoSorb (or both)? Presented at “the trinity of COVID-19: Immunity, Inflammation and Intervention Webinar,” May 20th 2020.	Webinar presentation of retrospective patients	Spain	7		Yes	Yes	59
Moazami N	CytoSorb: First Clinical Experience in the USA. Presented at the “EuroELSO Virtual ECMO Day,” June 25th 2020	Webinar presentation of retrospective patients	USA	10 vs 10	Yes		Yes	65
Nassiri AA	Blood Purification with CytoSorb in Critically Ill COVID-19 Patients: A Case Series of 26 Patients. Artif Org 2021; epub	Retrospective case series	Iran	26	Yes	Yes	Yes	66
Peng Z	Safety and Feasibility of CytoSorb Therapy in COVID-19 Patients–the Wuhan Experience. Presented at the “Cytokine Adsorption in Severely Ill COVID-19 Patients” Webinar. April 16th 2020.	Webinar presentation of retrospective patients	China	10	Yes	Yes	Yes	69
Nierhaus A	Rationale for the Use of CytoSorb in COVID-19 Patients. Presented at the “7th International CytoSorb Users' Meeting” Webinar. 29th Oct 2020	Webinar presentation of preliminary RCT results	Germany	5 vs 5	Yes		Yes	70
Lopez-Almarez J	CytoSorb Therapy in COVID-19 Patients. Experiences from Latin America. Presented at the “7th International CytoSorb Users' Meeting” Webinar. 29th Oct 2020	Webinar presentation of retrospective patients	Latin America	25	Yes	Yes	Yes	71
Riva I	Its More than Cytokine Removal, Presented at the “Cytokine Adsorption in Severely Ill COVID-19 Patients”	Webinar presentation of retrospective patients	Italy	11 vs 10		Yes	Yes	68

## Data Availability

The unpublished data used to support the findings of this study are available from the corresponding author upon request.
